# Expanded Vermiculite/D-Mannitol as Shape-Stable Phase Change Material for Medium Temperature Heat Storage

**DOI:** 10.3390/ma16186101

**Published:** 2023-09-07

**Authors:** Xifeng Lv, Chaoqun Fan, Ying Han, Xiaojin Tang, Changwei Zhang, Di Cai, Huidong Chen

**Affiliations:** 1 College of Chemistry and Chemical Engineering, Tarim University, Alar 843300, China; lvning7431@163.com (X.L.); 15071250977@163.com (C.F.); 2 SINOPEC Research Institute of Petroleum Processing, Beijing 100083, China; tangxj.ripp@sinopec.com; 3 National Energy R&D Center for Biorefinery, Beijing University of Chemical Technology, Beijing 100029, China; 13071172985@163.com (C.Z.); chenhdbuct@163.com (H.C.); 4 High-Tech Research Institute, Beijing University of Chemical Technology, Beijing 100029, China

**Keywords:** expanded vermiculite, D-mannitol, shape-stable phase change materials, heat storage

## Abstract

Aiming to promote the application of D-mannitol in the field of phase change thermal storage, obstacles, including low thermal storage efficiency and high supercooling, should be properly disposed of. The adoption of adaptable and low-cost supporting materials to make shape-stable phase change materials (ss-PCMs) affordable is a primary solution to solve the above shortcomings. In this study, high-performance ss-PCM for effective medium-temperature heat storage was prepared using expanded vermiculite as the support for D-mannitol preservation. Among the three candidates that treated the raw vermiculite by dilute acid, calcination, and microwave heating, the calcinated expanded vermiculite (CV) was characterized as the most suitable one. After impregnating D-mannitol into the CV carrier by vacuum, a melting enthalpy of 205.1 J/g and a crystallization enthalpy of 174.1 J/g were achieved by the as-received CV/D-mannitol ss-PCM. Additionally, the supercooling of the ss-PCM was reduced to 45.6 °C. The novel CV/D-mannitol ss-PCM also exhibited excellent reusability and stability. All the findings indicate that the abundant and inexpensive CV exhibited great potential as the supporting material for D-mannitol-based ss-PCMs, which allow effective waste heat recovery and temperature regulation.

## 1. Introduction

In facing the increasing unsustainable fossil fuel consumption and the large amounts of GHG emission, it is crucial to develop renewable energy systems for carbon peaking and carbon neutrality goals [1]. However, the rapid growth of the solar energy and wind energy industries has long been hindered by the inherent challenge of fluctuating energy supplies [2,3]. One of the most practical strategies in responding to the above shortcomings is the application of phase change materials (PCMs) for energy storage. In typical processes, the unstable supplied renewable energies could be transferred into thermal energy for storage and are released by transitions in the phase of the PCMs when needed [4]. Due to the excellent management of thermal energy, the PCMs are widely used in building energy efficient, solar thermal energy recovery, industrial waste heat recovery, and thermal insulation materials.

D-mannitol is a renewable biochemical that possesses high heat storage density and stability, which can be potentially used as a PCM for medium-temperature energy storage [5,6]. However, the realistic application of D-mannitol-based PCMs is always limited by the high degree of supercooling and the leakage issues [7]. To improve the PCM performances of D-mannitol, the primary approach is the proper encapsulation of D-mannitol into a micro-mesoporous scaffold material that relies on both microscopic confinement and macroscopic impregnation [8], forming a shape-stable phase change material (ss-PCM). Typically, the ss-PCMs’ performances are highly dependent on the structural characteristics of the supporting material [9]. At high temperatures, the liquid-state PCMs are easily trapped within the microporous structure by capillary forces as well as the intermolecular interactions, while the multiple-direction heat transfer would be promoted by the macropores of the enriched three-dimensional structure [10].

In the past decades, extensive research has been conducted for continuous exploration of the porous supporting materials candidates for the preservation of PCMs [11]. Among the candidate PCMs for supporting materials, mineral-based supports have always possessed the advantages of tunable structure, high chemical stability, affordability, and scientific endorsement. More importantly, mineral-based supports can be adjusted to meet specific requirements, such as resistance to chemical reactions and degradation [12]. For instance, Luo et al. prepared paraffin-based ss-PCMs using different origins of sepiolite as the supports [13]. The surface area of the acid-treated sepiolite reached 105.35 m^2^/g, and the relative enthalpy efficiency of the as-formed ss-PCMs was 94.45%. In another work, Zhang et al. designed mica-stabilized polyethylene glycol (PEG) ss-PCMs [14]. A maximum load of 46.24% was received, while the melting enthalpy and crystallization enthalpy were 77.75 J/g and 77.73 J/g, respectively.

Vermiculite is a silicate mineral that consists of a layered structure [15]. Due to its unique properties, vermiculite can be expanded by heating or chemical modification. After expansion, the volume of the raw vermiculite could be significantly increased, while the large pore structures provide ample space [16]. Although some previous studies have investigated the possibility of using vermiculite as the support material, there is still a lack of studies focused on developing the corresponding ss-PCMs for medium-temperature heat storage. Further, the effect of expansion mode and the state of vermiculite on the performances of the ss-PCM have not been fully explored. 

In this study, different expanding strategies, including acid treatment, calcination, and microwave heating, were conducted to generate a series of expanded vermiculites with different structural properties. Then, the expanded vermiculites were used as the supporting materials for the preservation of D-mannitol and used as the ss-PCMs. The moderately swollen vermiculite carriers are expected to facilitate the process of applying D-mannitol for efficient heat storage in the medium-temperature domain. This study adds new possibilities for vermiculite to develop advanced materials for sustainable energy storage applications.

## 2. Experimental

### 2.1. Materials

Vermiculite was purchased from Xinlong Vermiculite Co., Yuli, China. D-mannitol (98 wt%) and citric acid (90 wt%) were obtained from Macklin Co., Ltd., Shanghai, China. H_2_O_2_ (30%, *v*/*v*) was purchased from Tianjin Fuchen Chemical Reagent Co., Ltd., Tianjin, China.

### 2.2. Preparation of Expanded Vermiculite

To prepare the acid-treated expanded vermiculite, the raw vermiculite was first sieved to obtain particles with an approximate diameter of 2 mm. Then, the sieved vermiculite was dried out at 60 °C. In parallel, 4.0 g of citric acid was dissolved into 50 mL of H_2_O_2_ solution (15% (*v*/*v*)). Next, 10.0 g of dried vermiculite was added to the mixture, and the slurry stood for 24 h at 30 °C. Then, after washing with deionized water until it reached a neutral pH (~7), the solid fraction was collected and dried, which was defended as the acid-modified vermiculite (AV). The calcinated expanded vermiculite (CV) was prepared by heating the raw material in a muffle furnace at 800 °C for 5 min. The microwave-expanded vermiculite (MV) was obtained by heating the vermiculite in a microwave at 800 W for 10 min.

### 2.3. Preparation of the D-mannitol/Expanded Vermiculite as Shape-Stable Phase Change Materials

**Figure 1:**
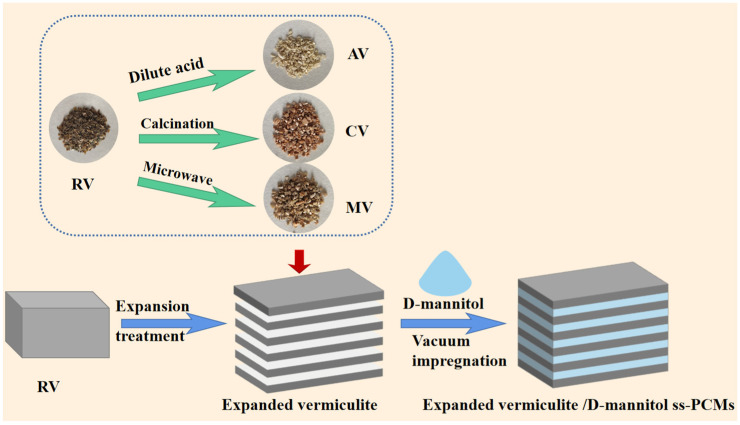
Flowchart for the preparation of expanded vermiculite/D-mannitol as ss-PCM.

The ss-PCMs were prepared using a vacuum impregnation strategy (Figure 1). In a typical process, 1.0 g of the expanded vermiculite, including AV, CV, and MV, were weighed and placed in a beaker. Then, 5 g of D-mannitol was placed on a copper sieve, which was positioned over the beaker. The beakers were subsequently placed in a vacuum oven at 180 °C for 30 min. Afterward, any excess D-mannitol on the surface of the ss-PCMs was removed by filtration. Finally, the ss-PCMs were named AV/D-mannitol, CV/D-mannitol, and MV/D-mannitol based on the types of expanded vermiculites used.

### 2.4. Characterization

Scanning electron microscopy (SEM, Hitachi SU1510, Tokyo, Japan) was used to characterize the morphology of the expanded vermiculites and the corresponding ss-PCM. The crystal structure of D-mannitol was investigated by X-ray diffraction (XRD, Rigaku, Tokyo, Japan, D/Max 2500 VB2 + PC) with scan 2*θ* values ranging from 5° to 80° and a scan rate of 5°/min. The chemical functional diagram of the ss-PCMs and the compatibility of the carrier with D-mannitol were tested using a Fourier transform infrared spectroscopy (FTIR, Thermo Fisher Nicolet 6700, Waltham, MA, USA) in the wave range of 4000–400 cm^−1^. Brunauer-Emmett-Teller (BET, Micromeritics ASAP 2460, Cambridge, MA, USA) was used to characterize the specific surface area and pore size distribution of the expanded vermiculite. The absorption and release of heat and the phase transition temperatures were analyzed by a differential scanning calorimeter (DSC, Switzerland Mettler DSC1, Greifensee, Switzerland). The heating/cooling rate was 5 °C/min, and the testing range was from room temperature (~25 °C) to 180 °C, followed by hold at 180 °C for 3 min. The thermal stability of the specimens in an N_2_ atmosphere was tested by thermogravimetric analysis (TGA, Switzerland Mettler, TGA/DSC3+) with a scanning temperature range of 30–800 °C, and a ramping rate of 5 °C/min. The ss-PCM specimens were placed in an oven at 180 °C and observed for melting and leakage.

## 3. Results and Discussion

### 3.1. Structure and Morphology of the Expanded Vermiculites as the Supporting Material

**Figure 2:**
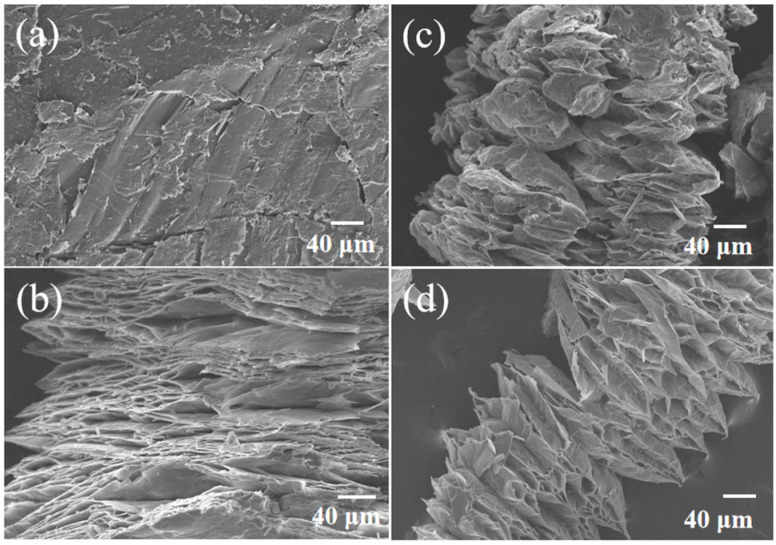
SEM of the (**a**) raw vermiculite, (**b**) AV, (**c**) CV, and (**d**) MV.

Before being used as a supporting material for the D-mannitol based ss-PCM, the expanded vermiculites specimens that were treated by different methods were characterized. As shown in Figure 2a, the original vermiculite had a dense stacked structure with fewer voids. However, after acidification, an obvious laminated structure appeared in the SEM image of AV [17], though the layer spacing was relatively low (Figure 2b). Compared with AV, calcination and microwave treatment were more effective in expanding the montmorillonite (Figure 2c,d), thus forming larger interlayer spacing.

**Figure 3:**
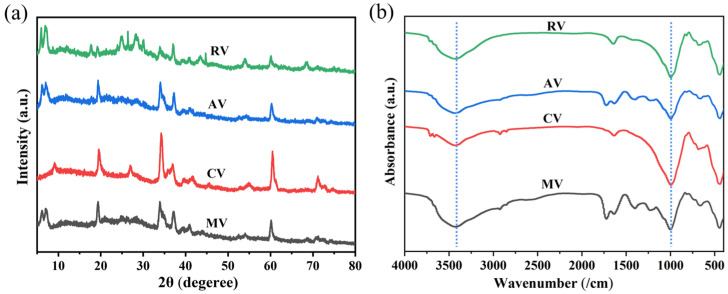
(**a**) XRD and (**b**) FTIR spectrums of the raw and treated vermiculite.

The XRD patterns in Figure 3a indicate that the initial composition of the supports consisted of vermiculite, auriculite-vermiculite, and auriculate [18]. After the pretreament of the raw vermiculite, changes were observed in the diffraction peak corresponding to the vermiculite (001) crystal plane at 8.9 degrees. The intensity of the peak was found to be reduced in the patterns of AV and MV, suggesting a weakening of the crystal faces [19]. Furthermore, in the CV pattern, the vermiculite (001) peak was nearly absent. This indicates that the vermiculite structure in AV and MV was not fully separated, and the organized structure of vermiculite was disrupted in the CV specimen. Additionally, a distinct diffraction peak at 27.5° corresponding to the crystalline plane of gold mica (003) was also observed in the CV pattern [20], suggesting that the gold mica did not undergo expansion during the treatment [21]. As can be seen from the FTIR spectrums (Figure 3b), the absorption peak at 3451 cm referred to the stretching vibrations of O-H in H_2_O and the peak at 1009 cm referred to the Si-O-Si and Si-O-Al functional bonds, which always appeared no matter the raw vermiculite treatment or not [22], suggesting the functional groups were not significantly changed after pretreatment.

**Figure 4:**
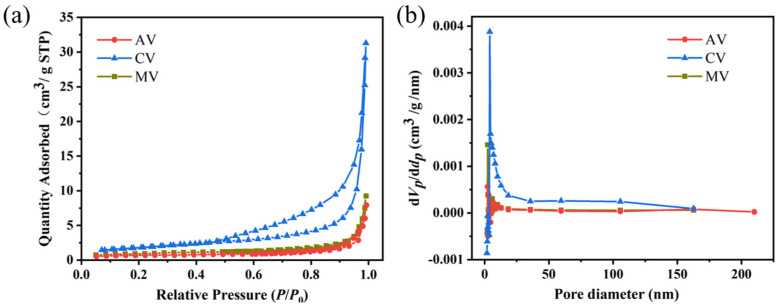
(**a**) N_2_ adsorption-desorption isotherms and the (**b**) pore size distribution of the expanded vermiculites after treatment.

The nitrogen adsorption and desorption of the expanded vermiculites after treatment were further tested. As can be seen from Figure 4a, all the curves for the expanded vermiculites at a low temperature were the type-IV curves [23], and the pore size distribution was also wider (Figure 4b). Among the specimens, the CV exhibited the largest specific surface area of 12.7 m^2^/g and the largest average pore size of 27.5 nm (Table 1). As was demonstrated in the literature, the presence of both macro- and nano-scale pore structures in CV would be conducive to improving the thermal properties of D-mannitol in the nanoporous restricted domain [9]. This hypothesis will be elaborated on in the following sections.

### 3.2. Morphology and Chemical Compatibility of the Shape-Stable Phase Change Materials

**Figure 5:**
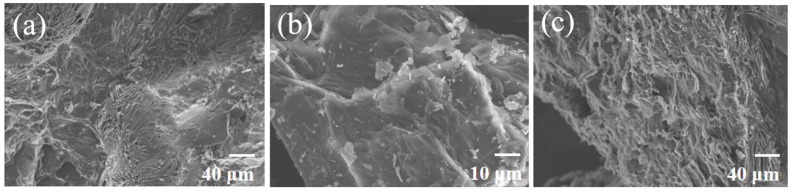
SEM image of (**a**) AV/D-mannitol, (**b**) CV/D-mannitol, and (**c**) MV/D-mannitol ss-PCMs.

The chemical compatibility of the specimens with D-mannitol is of significance in maintaining the stability of the ss-PCM [24]. Figure 5a shows a fuzzy boundary between AV and D-mannitol at the interface. In contrast, Figure 5c demonstrates complete adsorption of D-mannitol between the two layers of MV, resulting in even blurrier interfaces. Additionally, Figure 5b reveals a tightly arranged stacking pattern of the carrier and D-mannitol, suggesting that the impregnation of D-mannitol is uniform and adequate.

**Figure 6:**
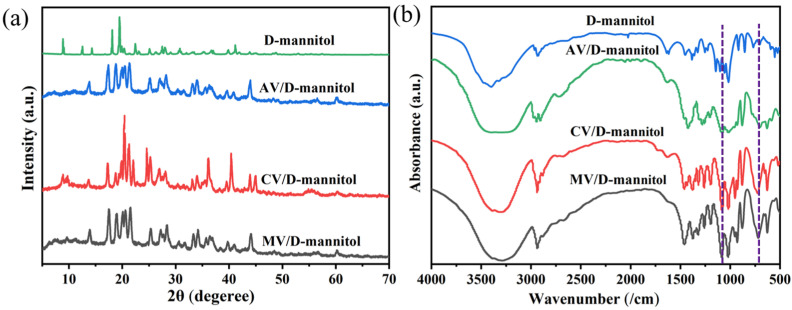
(**a**) XRD and (**b**) FTIR spectra of D-mannitol and ss-PCMs.

The XRD patterns of the pure D-mannitol and the corresponding ss-PCMs are shown in Figure 6a. Generally, the diffraction peaks of D-mannitol were consistent with the literature [25]. The diffraction peaks of D-mannitol can be observed in the ss-PCMs supported by the expanded vermiculites, inferring that there were no chemical changes in the D-mannitol after impregnation. In Figure 6b, the absorption peaks of D-mannitol at 1080 cm corresponded to the variable angle vibration of the alcohol hydroxyl group, while the peak at 700 cm was the a-type glycosidic bond [26]. These peaks were also detected in all of the tested ss-PCM specimens. Therefore, D-mannitol was bound to the supporting material by physical adsorption with good compatibility.

### 3.3. Characterization of the Phase Change Performances

Thermal storage capacity (*R*) and the effectiveness of latent heat storage (*E*) are the two decisive thermal performance indicators for thermal storage by ss-PCMs [27], which are related to the enthalpy of melting and crystallization of ss-PCMs. The enthalpies of melting (Δ*H_m_*) and crystallization (Δ*H_c_*) can be determined by calculating the integral area of the peaks in DSC curves. Phase transition temperatures can be determined by examining the baseline and the tangent intersects of the curves. *R* and *E* can be calculated by the following equations.

(1) $$R=\frac{\Delta H_{m,\mathrm{ss-PCM}}}{\Delta H_{m,\mathrm{D-mannitol}}}\times 100\%$$

(2) $$E=\frac{\Delta H_{m,\mathrm{ss-PCM}}+\Delta H_{c,\mathrm{ss-PCM}}}{\Delta H_{m,\mathrm{D-mannitol}}+\Delta H_{c,\mathrm{D-mannitol}}}\times 100\%$$

where Δ*H_m_*_,*D-mannitol*_ and Δ*H_c_*_,*D-mannitol*_ are the latent heat of D-mannitol in the melting and crystallization processes, respectively. Δ*H_m_*_,*ss-PCM*_ and Δ*H_c_*_,*ss-PCM*_ are the melting enthalpy and crystallization enthalpy of ss-PCMs, respectively.

**Figure 7:**
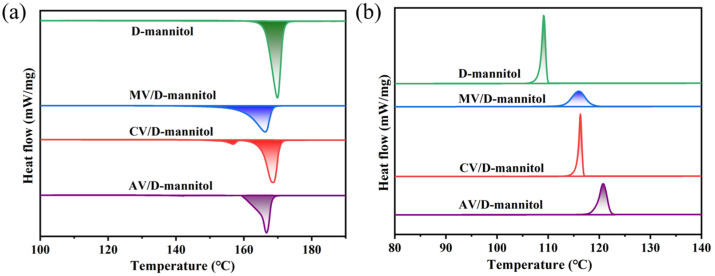
DSC curves of the pure D-mannitol and the ss-PCMs (AV/D-mannitol, CV/D-mannitol, and MV/D-mannitol) for (**a**) the melting and (**b**) the solidification processes.

Figure 7 shows the melting and crystallization progresses of pure D-mannitol and ss-PCMs, and the latent heat storage properties are shown in Table 2. Among the tested groups, the CV/D-mannitol possessed the highest Δ*H_m_* (195.9 J/g) and Δ*H_c_* (170.5 J/g), which may likely be due to the large specific surface area and pore structures of CV (Figure 4). The impressive *R* value of CV/D-mannitol (71.7%) reflects its superior thermal storage capacity. Furthermore, CV/D-mannitol also exhibited the highest *E* value (74.9%) among the tested ss-PCMs with different expanded vermiculites.

The degree of supercooling (Δ*T*) can be calculated from the difference between the melting temperature (*T_m_*) and the temperature of crystallization (*T_c_*): Δ*T* = *T_m_* − *T_c_*. According to the results shown in Table 2, all the texted expanded vermiculite supports for ss-PCMs could greatly improve the supercooling of D-mannitol. Attractively, due to the good ion exchange capacity caused by the slight destruction of the interlayer structure after microwave treatment, the heterogeneous nucleation of liquid D-mannitol molecules can be improved. Consequently, the supercooling of D-mannitol was significantly reduced to 40.8 °C in the MV/D-mannitol group. For the case that uses CV as the support for ss-PCMs, the rapid expansion of vermiculite by high-temperature calcination could obviously destroying the layered structure (this can be confirmed by the results in Figure 2, Figure 3 and Figure 4 and Table 1). Additionally, the calcination process also transformed the interlayers of vermiculite into a glass phase. These changes in the structure and composition of vermiculite altered the charge balance, which further resulted in a relatively low cation exchange capacity [28]. Fortunately, in the CV/D-mannitol group, it was observed that D-mannitol has a slightly higher supercooling temperature (45.6 °C), but possessed higher thermal storage capacity (*R)* and efficiency (*E)*. Through analyzing the enthalpy of phase change and conducting a comprehensive comparison, CV is selected as the the optimal support material for impregnating D-mannitol among the candidates. Table 3 summarizes the current progress of ss-PCMs using expanded vermiculite as the supporting material. CV/D-mannitol makes it more competitive with other ss-PCMs with a relatively high enthalpy of melting and enthalpy of crystallization, suggesting that the introduction of expanded vermiculite as a support skeleton was a suitable method for the medium-temperature ss-PCMs with good thermal storage properties.

**Figure 8:**
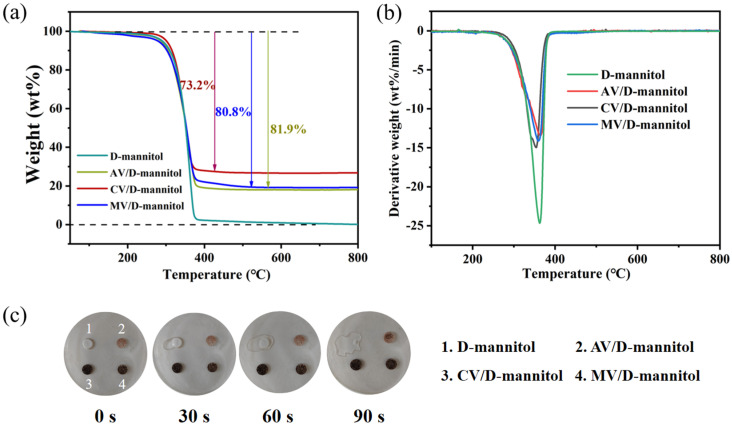
(**a**) TG and (**b**) DTA thermograms of the pure D-mannitol and the ss-PCMs, and the (**c**) photograph of the leakages of D-mannitol and ss-PCMs.

The thermal stability of CV/D-mannitol ss-PCM was further investigated. As shown in Figure 8a, there was almost no weight loss of the CV/D-mannitol before 283.8 °C, and it gradually decomposed when the temperature became higher. A maximum decomposition temperature of 362.8 °C was observed (Figure 8b), indicating that the ss-PCMs exhibited good thermal stability. At 400 °C, there were only 2.2 wt% of D-mannitol remaining, indicating a high degree of decomposition. The thermogravimetric (TG) curves of the ss-PCMs using expanded vermiculites as the support showed similar mass loss characteristics to pure D-mannitol. The thermal decomposition temperatures of AV/D-mannitol, CV/D-mannitol, and MV/D-mannitol were all above 280 °C, and the weight losses at equilibrium were 81.9%, 73.2% and 80.8%, respectively. The leaking behaviors of the pure D-mannitol and the ss-PCMs that were supported by expanded vermiculites were compared in Figure 8c. When the temperature of the surrounding environment was maintained at 180 °C, pure D-mannitol melted completely within 90 s. However, there was no noticeable leakage of D-mannitol from the tested ss-PCMs, indicating the excellent PCM preservation ability provided by the expanded vermiculites.

**Figure 9:**
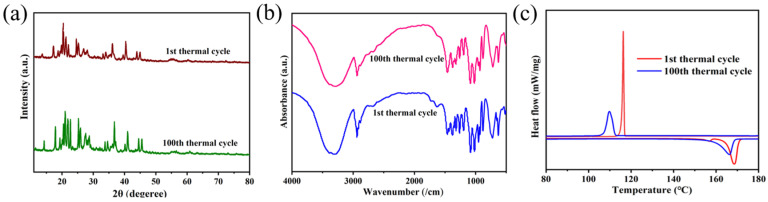
(**a**) XRD patterns, (**b**) FTIR spectra, and (**c**) DSC curves of the CV/D-mannitol before and after 100 cycles of heating and cooling.

In order to further analyze the long-term stability of the novel CV/D-mannitol ss-PCMs, we performed 100 cycles of heating and cooling progression. The XRD patterns and FTIR spectrum of the fresh and used ss-PCMs are shown in Figure 9a,b. Overall, the peak positions and shapes were almost unchanged, indicating good stability of the ss-PCMs. After 100 cycles of operation, Δ*H_m_* and Δ*H_c_* only decreased by 4.8% and 4.2%, respectively (Figure 9c and Table 4). Therefore, the CV/D-mannitol ss-PCMs possess good recyclability and have the potential to be applied in realistic long-term medium-temperature heat storage processes.

## 4. Conclusions

The expanded vermiculite obtained from raw vermiculite through dilute acid treatment, calcination, and microwave heating can be used as supporting materials for ss-PCMs. Among them, CV exhibits superior performances in impregnating D-mannitol compared to other materials. The CV/D-mannitol exhibited Δ*H_m_* of 195.9 J/g and Δ*H_c_* of 170.5 J/g, which also showed the capability to reduce the supercooling of D-mannitol to 45.6 °C, effectively alleviating the heat release delay phenomenon. Even after 100 cycles of heating/cooling cycles, the CV/D-mannitol can still maintain a high phase change enthalpy, showing good durability and suitability for long-term use. Therefore, CV/D-mannitol showed great promise in medium temperature heat storage.

## Figures and Tables

**Table 1:** Specific surface area and the average pore width of the expanded vermiculites.

	Sample		
	AV	CV	MV
Specific surface area (m^2^/g)	7.4	12.7	8.6
Average pore width (nm)	16.5	27.5	16.8

**Table 2:** Latent heat storage properties of pure D-mannitol and ss-PCMs.

Sample	Δ*H_m_* (J/g)	*T_m_* (°C)	Δ*H_c_* (J/g)	*T_c_* (°C)	Δ*T*	*R*	*E*
D-mannitol	273.1	167.6	215.9	113.7	53.9	-	-
AV/D-mannitol	185.9	165.3	166.8	123.2	42.1	68.1	72.1
CV/D-mannitol	195.9	165.2	170.5	126.6	45.6	71.7	74.9
MV/D-mannitol	176.7	163.2	160.1	122.4	40.8	64.7	68.9

**Table 3:** Current advances of the ss-PCM using expanded vermiculite as supports.

Sample	Δ*H_m_* (J/g)	*T_m_* (°C)	Δ*H_c_* (J/g)	*T_c_* (°C)	References
EV/n-octadecane	142.0	26.1	126.5	24.9	[29]
Fatty acid/EV/expanded graphite	71.5	25.6	69.4	24.9	[30]
Paraffin/EV	110.0	48.0	113.1	52.5	[31]
PEG/AEVM-3	154.8	60.1	136.2	29.2	[32]
C-EVM/P	208.0	21.2	203	−3.40	[33]
CV/D-mannitol	195.9	165.2	170.5	126.6	This work

**Table 4:** Thermal properties of the CV/D-mannitol ss-PCM before and after 100 heating/cooling cycles.

Cycles	Δ*H_m_* (J/g)	*T_m_* (°C)	Δ*H_c_* (J/g)	*T_c_* (°C)
1st	195.9	165.2	170.5	126.6
100th	186.5	160.4	163.4	118.7

## Data Availability

All data ate contained within the article.
